# R1p Dispersion in White Matter Correlates with Cognitive Impairment in Older Adults

**DOI:** 10.1093/geroni/igac059.1375

**Published:** 2022-12-20

**Authors:** Fatemeh Adelnia, Taylor Davis, Lealani Mae Acosta, Amanda Puckett, Feng Wang, Zhongliang Zu, Kevin Harkins, John Gore

**Affiliations:** Vanderbilt University Medical Center, Nashville, Tennessee, United States; Vanderbilt University Medical Center, Nashville, Tennessee, United States; Vanderbilt University Medical Center, Nashville, Tennessee, United States; Vanderbilt University Medical Center, Nashville, Tennessee, United States; Vanderbilt University Medical Center, Nashville, Tennessee, United States; Vanderbilt University Medical Center, Nashville, Tennessee, United States; Vanderbilt University Medical Center, Nashville, Tennessee, United States; Vanderbilt University Medical Center, Nashville, Tennessee, United States

## Abstract

Alzheimer‘s disease is the most frequent form of dementia in older adults and has a total estimated worldwide cost that rises to $2 trillion by 2030. Much previous neuroimaging research in AD has focused on the roles of amyloid and tau proteins using PET, but there have also been several studies that have implicated microvascular changes as an early indicator of damage related to later dementia. Here, we developed and refined a non-invasive 3D-R1ρ dispersion imaging technique using different locking fields to quantify microvasculature changes within brain tissues in persons with mild cognitive impairment (MCI) compared to healthy controls. The fractional difference in R1ρ comparing different locking fields provides a unique way of characterizing changes in the geometry and structure of microvasculature. After providing informed consent, 40 adults aged 62 to 82 years (n=17MCI) underwent cognitive assessments and MRI scan at 3T. We found the fractional change in R1ρ of the whole brain white matter is significantly greater in persons with MCI, and the correlation remained significant (ß=-0.4, p-value=0.01) after introducing age (ß=0.2, p-value=0.2) and sex (ß=-0.1, p-value=0.5) as covariates. The white matter hypertonicity lesion volume measured from conventional MRI was also correlated with the health status (p-value < 0.05); however, the size of the regression coefficient was substantially smaller (53% lower), and it was no longer significant (p-value=0.14) after adjusting for age and sex. This work establishes a new non-invasive method that can potentially characterize changes in microvasculature anatomy with the progression of cognitive impairment regardless of an age effect.

